# Piceatannol and Its Metabolite, Isorhapontigenin, Induce SIRT1 Expression in THP-1 Human Monocytic Cell Line

**DOI:** 10.3390/nu6114794

**Published:** 2014-10-30

**Authors:** Shinpei Kawakami, Yosuke Kinoshita, Hiroko Maruki-Uchida, Koji Yanae, Masahiko Sai, Tatsuhiko Ito

**Affiliations:** Research Institute, Morinaga and Company Limited, 2-1-1 Shimosueyoshi, Tsurumi-ku, Yokohama 230-8504, Japan; E-Mails: s-kawakami-jf@morinaga.co.jp (S.K.); y-kinoshita-jg@morinaga.co.jp (Y.K.); k-yanae-ja@morinaga.co.jp (K.Y.); m-sai-ia@morinaga.co.jp (M.S.); t-ito-jj@morinaga.co.jp (T.I.)

**Keywords:** piceatannol, isorhapontigenin, resveratrol, sirtuin 1, THP-1

## Abstract

Piceatannol is a phytochemical that is present in large amounts in passion fruit (*Passiflora edulis*) seeds, and is an analog of resveratrol. Recently, the absorption and metabolism of piceatannol were investigated in rats, and isorhapontigenin, *O*-methyl piceatannol, was detected as a piceatannol metabolite in rat plasma. To elucidate the function of piceatannol and its metabolites, we investigated the expression of sirtuin 1 (SIRT1) in THP-1 monocytic cells after treatment with piceatannol and its metabolites, and compared their effects with those of resveratrol and its metabolites. Piceatannol and resveratrol upregulated the expression levels of *SIRT1* mRNA and SIRT1 protein. An extract of passion fruit seeds, which contained high levels of piceatannol, also upregulated *SIRT1* mRNA expression. As for the metabolites, isorhapontigenin upregulated *SIRT1* mRNA expression, whereas resveratrol glucuronides and sulfate did not affect *SIRT1* expression. These findings indicate that after intake of piceatannol, not only piceatannol itself, but also its metabolite, isorhapontigenin, contributed to the upregulation of SIRT1 expression.

## 1. Introduction

Piceatannol, an analog of resveratrol, is a polyphenolic stilbene phytochemical that has anti-inflammatory, anti-proliferative, and anti-adipogenesis activities [1,2]. Our previous studies demonstrated that piceatannol is present in large amounts in passion fruit (*Passiflora edulis*) seeds [3]. We have also demonstrated that piceatannol displays a wide spectrum of biological activities, such as acute vasorelaxant effects in thoracic aorta [4], upregulation of endothelial nitric oxide synthase in endothelium [5], inhibition of melanogenesis, promotion of collagen synthesis [3], and protection of skin from UV-B irradiation [6].

To elucidate the bioavailability and metabolism of piceatannol, we investigated the absorption and metabolism of piceatannol in rats [7]. After intragastric administration of piceatannol, isorhapontigenin, an *O*-methyl piceatannol metabolite, was detected in the intact form in rat plasma. The area under the plasma concentration curve (AUC) for isorhapontigenin was roughly one-third of the AUC for piceatannol. Rhapontigenin, another *O*-methyl piceatannol metabolite, was detected only in conjugated form. On the other hand, resveratrol metabolites were found only as glucuronide and sulfate conjugates. It is important to know whether these metabolites, as well as intact compounds, exert any biological activity. There have been several studies on the function of resveratrol metabolites [8,9]; however, little is known about the biological activity of piceatannol metabolites.

Sirtuin proteins are NAD^+^-dependent deacetylases that have been linked to the regulation of life span in response to caloric restriction [10]. The function of sirtuin 1 (SIRT1) has been well studied, and activation of SIRT1 may be beneficial in age-related disorders such as type 2 diabetes and neurodegenerative diseases [11]. In peripheral blood mononuclear cells (PBMCs), the expression levels of the SIRT1 were lower in subjects with metabolic syndrome than in subjects without metabolic syndrome [12]. Similarly, low SIRT1 expression was observed in insulin-resistant subjects [12]. It was also reported that *SIRT1* mRNA expression levels in PBMCs were low in subjects with coronary artery disease [13]. These finding indicated that SIRT1 expression in PBMCs was negatively correlated with metabolic syndrome, insulin sensitivity, and atherosclerosis. Therefore, maintaining SIRT1 levels or stimulating the upregulation of SIRT1 expression could be beneficial in the treatment of a variety of chronic diseases.

Dietary polyphenols, such as resveratrol, have been shown to activate SIRT1 in a variety of models [14,15,16]. Resveratrol has been shown to upregulate the expression of SIRT1, and that resulted in the regulation of energy expenditure and a decrease in fat accumulation [17,18]. In THP-1 cells, a human monocytic cell line, resveratrol also upregulated *SIRT1* mRNA expression under high-glucose conditions [19]. On the other hand, piceatannol has been reported to enhance the deacetylase activity of SIRT1 *in vitro* [20]. However, little is known about the effect of piceatannol or its metabolites on SIRT1 expression.

In the present study, we investigated the expression of SIRT1 in THP-1 human monocytic cell line after treatment with piceatannol, and compared the effects of piceatannol with those of resveratrol. We also investigated the effect of passion fruit seed extract, which contains high levels of piceatannol, on SIRT1 expression, and compared its activity to that of purified piceatannol. Furthermore, the effects of metabolites of piceatannol and resveratrol on SIRT1 expression were investigated.

## 2. Experimental Section

### 2.1. Materials

**Figure 1:**
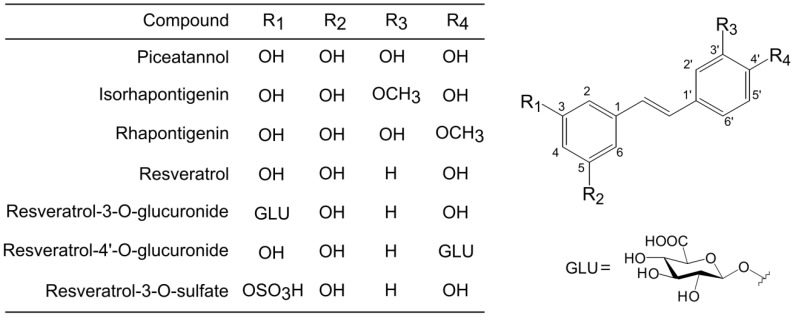
Chemical structures of piceatannol, resveratrol, and their metabolites.

Piceatannol, resveratrol, isorhapontigenin, rhapontigenin were obtained from Tokyo Chemical Industry, Co., Ltd. (Tokyo, Japan). Resveratrol 3-*O*-glucuronide was obtained from Toronto Research Chemicals, Inc. (Ontario, Canada). Resveratrol-4′-*O*-glucuronide and resveratrol-3-*O*-sulfate were obtained from Bertin Pharma (Montigny le Bretonneux, France). Figure 1 shows the structure of all stilbenes used in this study. Fetal bovine serum was obtained from HyClone (Logan, UT, USA). RPMI 1640 medium and antibiotics were obtained from Life Technologies (Gaithersburg, MD, USA). All other reagents were obtained from Wako Pure Chemical Industries, Ltd. (Osaka, Japan).

### 2.2. Preparation of Passion Fruit Seed Extract

Passion fruit seeds were freeze-dried, milled, and extracted with 80% ethanol. After centrifugation, the supernatant was evaporated, and solvent was removed from the pellet by freeze-drying. The concentration of piceatannol in the lyophilized powder was measured using high-performance liquid chromatography (HPLC) analysis as previously described [7]. The powder contained piceatannol (85.4 μg/mg) as a primary polyphenol. The powder also contained 54.7 μg/mg scirpusin B, a dimer of piceatannol. We used this powder as passion fruit seed extract in this study.

### 2.3. Cell Culture and Treatment

Human THP-1 cells were grown at 37 °C in a humidified atmosphere containing 5% CO_2_ in RPMI 1640 medium containing 10% fetal bovine serum, 100 units/mL penicillin, 100 μg/mL streptomycin, and 1 mM sodium pyruvate. Cells were seeded in 6-well plates at 2 × 10^5^ cells per well and incubated for 2 to 24 h in cell culture media containing different concentrations of stilbene test compounds. Control cells were incubated with the same medium containing the amount of solvent (dimethyl sulfoxide, DMSO) used to dissolve the stilbene test compounds.

### 2.4. Determination of SIRT1 mRNA Expression

Determination of mRNA expression was performed as previously described [5]. Total RNA samples extracted from cells were reverse transcribed into complementary DNA, and real-time polymerase chain reactions (PCR) were performed in triplicate using a Light Cycler 480 Real-Time PCR system II (Roche Diagnostics, Mannheim, Germany). PCR primers used were forward (5′-ctccaaggccacggatag-3′) and reverse (5′-gccacagtgtcatatcatcca-3′) for SIRT1 and forward (5′-agccacatcgctcagacac-3′) and reverse (5′-gcccaatacgaccaaatcc-3′) for glyceraldehyde 3-phosphate dehydrogenase (GAPDH). Amplification conditions were 50 °C for 2 min, 95 °C for 10 min, followed by 45 cycles at 95 °C for 10 s and 60 °C for 25 s. *SIRT1* mRNA expression was normalized to *GAPDH* mRNA expression levels, after which relative *SIRT1* mRNA expression was determined in comparison with the levels in control cells.

### 2.5. Western Blotting Analysis of SIRT1 Protein Expression

THP-1 cells were lysed in ice-cold radio-immunoprecipitation assay (RIPA) buffer containing 1 mM phenylmethylsulfonyl fluoride (PMSF, Cell Signaling, Beverly, MA, USA). The lysates were sonicated and centrifuged at 20,400× *g* for 5 min at 4 °C. 25 μg of protein was separated by 7.5% SDS-PAGE and transferred to polyvinylidene fluoride membranes. The membranes were blocked using 5% skim milk in Tris-buffered saline containing 0.1% Tween 20 (TBST) for 1 h and incubated with mouse anti-SIRT1 antibodies (1:1000, Cell Signaling), or mouse anti-β-actin antibodies (1:5000, Sigma, St. Louis, MO, USA) overnight at 4 °C. Membranes were washed with TBST and incubated with horseradish peroxidase-conjugated anti-mouse IgG (1:2500 for SIRT1 detection and 1:20000 for β-actin detection, Cell Signaling) for 1 h at room temperature. Primary antibodies were diluted with 3% bovine serum albumin in TBST, and secondary antibodies were diluted with 5% skim milk in TBST. Visualization of immunoreactive bands was performed using the ECL Prime Western Blotting Detection System (GE Healthcare, Little Chalfont, UK). The intensity of each band was determined by ImageJ software (National Institutes of Health, Bethesda, MD, USA), and normalized to the intensity of the β-actin band.

### 2.6. Statistical Analysis

Data are presented as mean and standard deviation (S.D.). Statistical analyses were performed with one-way analysis of variance (ANOVA), followed by Tukey’s test for multiple comparisons using SPSS software (SPSS Inc., Tokyo, Japan.). A value of *p* < 0.05 was considered significant.

## 3. Results

**Figure 2:**
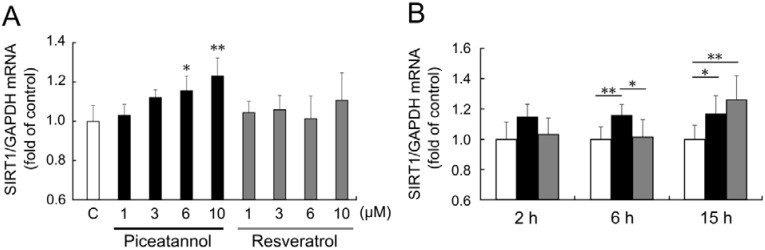
(**A**) THP-1 cells were treated with 0.1% dimethyl sulfoxide (DMSO, control; white bar), 1–10 μM piceatannol (black bars), or 1–10 μM resveratrol (gray bars) for 6 h. (**B**) THP-1 cells were treated with 0.1% DMSO (control, white bars), 6 μM piceatannol (black bars), or 6 μM resveratrol (gray bars) for 2–15 h. Induction of sirtuin 1 (*SIRT1*) mRNA expression was examined by quantitative PCR. The ratio of *SIRT1* to glyceraldehyde 3-phosphate dehydrogenase (*GAPDH*) were expressed as fold change normalized to control and expressed as means of five individual experiments + S.D. ** *p* < 0.01, * *p* < 0.05 (Tukey’s test). C: control.

To investigate the effect of piceatannol on *SIRT1* mRNA expression, we performed real-time PCR analysis. When THP-1 cells were treated with 1–10 μM piceatannol for 6 h, *SIRT1* mRNA expression was upregulated in a concentration-dependent manner (Figure 2A). On the other hand, treatment with resveratrol (1–10 µM) for 6 h did not show significant *SIRT1* upregulation.

Time-course of *SIRT1* mRNA expression after piceatannol and resveratrol stimulation was also investigated. Stimulation by 6 μM piceatannol for 2 h showed a slight upregulation of *SIRT1* mRNA expression levels, but the change was not statistically significant; however, stimulation for 6 and 15 h significantly upregulated *SIRT1* mRNA expression level (Figure 2B). Only stimulation with 6 μM resveratrol for 15 h upregulated *SIRT1* mRNA expression level, and that for 2 or 6 h did not affect *SIRT1* mRNA expression.

**Figure 3:**
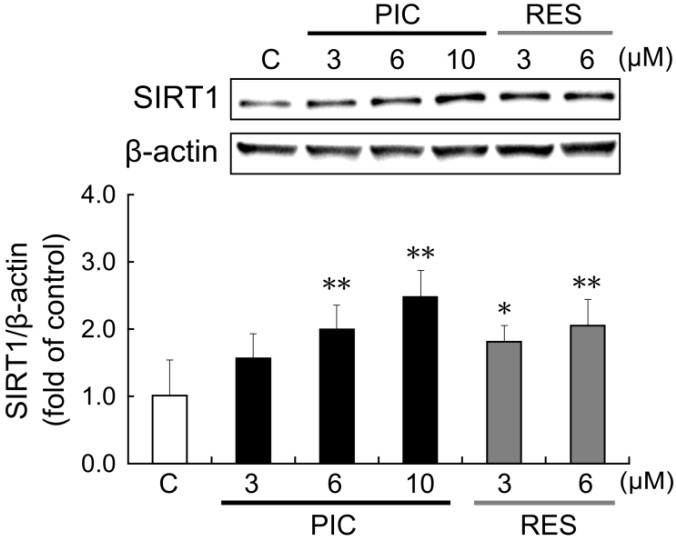
THP-1 cells were treated with 0.1% DMSO (control, white bars), 3–10 μM piceatannol (black bars), or 3–6 μM resveratrol (gray bars). After 24 h, SIRT1 protein induction was examined by western blot analysis. The ratio of the SIRT1 band to the β-actin band was determined and then expressed as fold of control. Data are shown as mean + S.D. (*n* = 6). The upper panel depicts a representative blot. ** *p* < 0.01, * *p* < 0.05 (Tukey’s test). C: control; PIC: piceatannol; RES: resveratrol.

To investigate the effects of piceatannol and resveratrol on SIRT1 protein, we performed western blot analysis. After 24 h treatment with piceatannol, SIRT1 protein expression increased in a concentration-dependent manner (Figure 3). SIRT1 protein level increased by 2.0- and 2.5-fold after administration of 6 and 10 μM piceatannol, respectively. Resveratrol also increased SIRT1 protein expression in a concentration-dependent manner, 1.8-fold at 3 μM and 2.0-fold at 6 μM. After 24 h treatment with 10 μM resveratrol, cell proliferation was strongly inhibited, therefore the data was not used.

**Figure 4:**
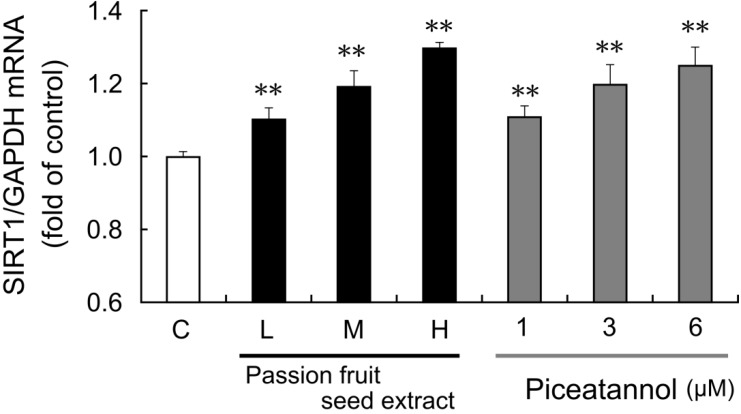
THP-1 cells were treated with 0.1% DMSO (C: control, white bars), passion fruit seed extract (black bars), or 1–6 μM piceatannol (gray bars) for 15 h. Low (L: 8.6 μg/mL), middle (M: 17.2 μg/mL), high (H: 28.6 μg/mL) concentration of extract contains 1, 3, 6 μM piceatannol, respectively. Induction of *SIRT1* mRNA expression was determined by quantitative PCR. The ratio of *SIRT1* to *GAPDH* was calculated and expressed as fold of control. Data are shown as the mean + S.D. (*n* = 5). ** *p* < 0.01 (Tukey’s test).

The effect of passion fruit seed extract on *SIRT1* mRNA expression in THP-1 cells was also examined. After 15 h exposure to passion fruit seed extract, *SIRT1* mRNA expression was upregulated in a concentration-dependent manner (Figure 4). Piceatannol reagents were prepared at a concentration equal to piceatannol containing in this extract, and both passion fruit seed extract and piceatannol upregulated *SIRT1* mRNA expression to the same extent.

**Figure 5:**
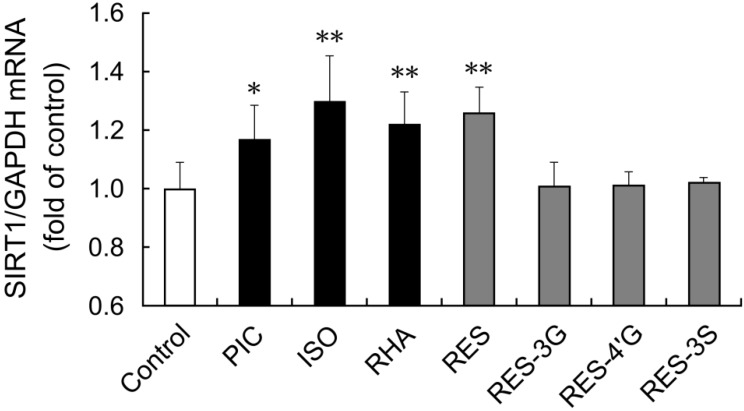
THP-1 cells were treated with 0.1% DMSO (control), or 6 μM of each stilbene for 15 h. Induction of *SIRT1* mRNA expression was examined by quantitative PCR. The ratio of *SIRT1* to *GAPDH* was determined and expressed as fold of control. Data are shown as the mean + S.D. (*n* = 3–7). ** *p* < 0.01, * *p* < 0.05 (Tukey’s test). Cont: control; PIC: piceatannol; ISO: isorhapontigenin; RHA: rhapontigenin; RES: resveratrol; RES-3G: resveratrol-3-*O*-glucuronide; RES-4′G: resveratrol-4′-*O*-glucuronide; RES-3S: resveratrol-3-*O*-sulfate.

We also examined whether other stilbenes affected SIRT1 expression in THP-1 cells. Isorhapontigenin and rhapontigenin are both forms of *O*-methyl piceatannol [7]. Resveratrol-3-*O*-glucuronide, resveratrol-4′-*O*-glucuronide and resveratrol-3-*O*-sulfate are metabolites of resveratrol. These conjugates have been detected in human plasma after oral administration of resveratrol [21]. After 15 h stimulation with isorhapontigenin or rhapontigenin, *SIRT1* mRNA expression was upregulated in THP-1 cells (Figure 5). In contrast, the glucuronide and sulfate metabolites of resveratrol did not affect *SIRT1* mRNA expression.

## 4. Discussion

In this study, we investigated the effects of stilbenes on SIRT1 expression in the THP-1 human monocytic cell line. We found that piceatannol and resveratrol increased *SIRT1* mRNA and SIRT1 protein expression. An extract of passion fruit seeds, which contain high levels of piceatannol, also increased SIRT1 expression. Furthermore, isorhapontigenin and rhapontigenin, *O*-methyl metabolites of piceatannol, also increased SIRT1 expression.

A notable characteristic of piceatannol is its methylated metabolites. In the present study, isorhapontigenin and rhapontigenin showed SIRT1 induction effects in THP-1 cells. After intragastric administration of piceatannol, isorhapontigenin was detected in both the intact and conjugated forms, and rhapontigenin was detected only in conjugated form [7]. Therefore, after piceatannol intake, isorhapontigenin could contribute to the upregulation of SIRT1 additively with intact piceatannol.

In previous studies, resveratrol was absorbed, rapidly metabolized, and, consequently, primarily found in the form of glucuronide and sulfate conjugates in plasma [22,23]. After oral administration of resveratrol in healthy volunteers, resveratrol-3-*O*-sulfate and resveratrol-3-*O*-glucuronide were the primary forms detected [21]. Studies have been published about the biological activity of resveratrol metabolites. Resveratrol conjugates have been reported to modulate inflammation pathways *in vitro* with similar efficacy to intact resveratrol [9], and to regulate adipokine expression and secretion in 3T3-L1 cells [8]. On the other hand, our results showed that resveratrol, but not its glucuronide and sulfate conjugates, upregulated SIRT1 expression. It has been reported that resveratrol increased forkhead box protein O1 (FoxO1)-dependent SIRT1 transcriptional activity [24]. However, the mechanism of stilbene-induced SIRT1 upregulation is not well understood. Spatial and structural changes in resveratrol by conjugation may affect activity for SIRT1 induction. Although the effects of glucuronide and sulfate conjugates of piceatannol on SIRT1 expression have not been investigated, these conjugates may also lack activity on SIRT1 induction, similar to the resveratrol conjugates.

In the present study, piceatannol and resveratrol upregulated SIRT1 expression to a similar extent. However, *in vivo*, the AUC for intact piceatannol was higher than that of intact resveratrol [7], and, in addition, a metabolite of piceatannol retained effects on upregulation of SIRT1. These observations suggest that piceatannol may be more effective than resveratrol in upregulating SIRT1 expression, owing to a higher concentration in plasma and the presence of an active metabolite.

The stilbenes were tested at 6 μM and significantly induced SIRT1 expression in this study. The initial serum piceatannol concentration after intragastric administration of 180 μmol/kg body weight was found to be 7.5 μM [7]. In addition, after oral intake of 2000 mg *trans*-resveratrol in human subjects, the maximum plasma concentration of trans-resveratrol reached 5.6 μM [25]. These data suggest that the concentration of stilbenes used in this study were in the range that was achievable *in vivo*.

Passion fruit is consumed throughout the world, and the seeds are often eaten together with the pulp in the natural state. The major polyphenol of passion fruit seed extract is piceatannol, and the content of piceatannol in passion fruit seeds is higher than that found in other plants, such as grapes [3]. In this study, passion fruit seed extract showed SIRT1-induction as did purified piceatannol. To evaluate the contribution of piceatannol to the effects of the extract, we compared the bioactivity of the extract with purified piceatannol. The result showed that the effect of passion fruit seed extract on *SIRT1* mRNA induction were similar to the effect of purified piceatannol. There are some other polyphenols in this extract, such as scirpusin B, a dimer of piceatannol [4]. However, purified scirpusin B from the extract did not show SIRT1-inducing effects (data not shown). Therefore, piceatannol is the main bioactive component responsible for the SIRT1-inducing effect of passion fruit seed extract.

It has been reported that inhibition of SIRT1 activity in THP-1 cells induces the expression of inflammation-related genes, such as tumor necrosis factor alpha and interleukin-6, through the activation of nuclear factor kappa B signaling [26]. Moreover, low SIRT1 levels were correlated with insulin resistance and obesity [12,13]. On the other hand, SIRT1 expression in PBMCs was upregulated in overweight subjects by calorie restriction [27]. This treatment resulted in significant decreases in body weight, body mass index, free fatty acid levels, fasting insulin, and inflammatory markers such as interleukin-6 and visfatin. Moreover, transgenic mice overexpressing SIRT1, when fed a high-fat diet, showed less lipid-induced inflammation and better glucose tolerance than non-transgenic mice [28]. These results suggest that upregulation of SIRT1 and high levels of SIRT1 expression have the potential to improve chronic inflammation, insulin resistance, and obesity. Although *in vivo* and clinical studies will be needed to evaluate the effects of piceatannol or passion fruit seed extract intake, ingestion of piceatannol may contribute to the treatment and prevention of various chronic diseases, such as inflammation, metabolic syndrome, and obesity, via upregulation of SIRT1.

## 5. Conclusions

Our study demonstrated that piceatannol and its metabolite isorhapontigenin upregulated *SIRT1* mRNA and SIRT1 protein in THP-1 monocytic cells. These results suggested that after intake of piceatannol, not only intact piceatannol but also its metabolite, isorhapontigenin, contributed to upregulation of SIRT1 expression. Given the stability in plasma of piceatannol in its intact form and activity of its methylated metabolite, piceatannol may be more effective than resveratrol in upregulating SIRT1 expression. Piceatannol may contribute to the treatment and prevention of various chronic diseases in the future.
